# Knowledge, attitudes, and practices of cardiovascular health care personnel regarding coronary CTA and AI-assisted diagnosis: a cross-sectional study

**DOI:** 10.7189/jogh.15.04103

**Published:** 2025-07-04

**Authors:** Shanshan Jiang, Lu Ma, Keqin Pan, Hongxia Zhang

**Affiliations:** 1 Department of Radiology, Tsinghua University Hospital, Beijing, China; 2 Department of Radiology, Beijing Bo’ai Hospital, China Rehabilitation Research Center, Beijing, China

## Abstract

**Background:**

Artificial intelligence (AI) holds significant promise for medical applications, particularly in coronary computed tomography angiography (CTA). We assessed the knowledge, attitudes, and practices (KAP) of cardiovascular health care personnel regarding coronary CTA and AI-assisted diagnosis.

**Methods:**

We conducted a cross-sectional survey from 1 July to 1 August 2024 at Tsinghua University Hospital, Beijing, China. Healthcare professionals, including both physicians and nurses, aged ≥18 years were eligible to participate. We used a structured questionnaire to collect demographic information and KAP scores. We analysed the data using correlation and regression methods, along with structural equation modelling.

**Results:**

Among 496 participants, 58.5% were female, 52.6% held a bachelor’s degree, and 40.7% worked in radiology. Mean KAP scores were 13.87 (standard deviation (SD) = 4.96, possible range = 0–20) for knowledge, 28.25 (SD = 4.35, possible range = 8–40) for attitude, and 31.67 (SD = 8.23, possible range = 10–50) for practice. Knowledge (*r* = 0.358; *P* < 0.001) and attitude positively correlated with practice (*r* = 0.489; *P* < 0.001). Multivariate logistic regression indicated that educational level, department affiliation, and job satisfaction were significant predictors of knowledge. Attitude was influenced by marital status, department, and years of experience, while practice was shaped by knowledge, attitude, departmental factors, and job satisfaction. Structural equation modelling showed that knowledge was directly affected by gender (β = −0.121; *P* = 0.009), workplace (*β = −*0.133; *P* = 0.004), department (*β = −*0.197; *P* < 0.001), employment status (*β = −*0.166; *P* < 0.001), and night shift frequency (*β =* 0.163; *P* < 0.001). Attitude was directly influenced by marriage (*β =* 0.124; *P* = 0.006) and job satisfaction (*β = −*0.528; *P* < 0.001). Practice was directly affected by knowledge (*β =* 0.389; *P* < 0.001), attitude (*β =* 0.533; *P* < 0.001), and gender (*β = −*0.092; *P* = 0.010). Additionally, gender (*β = −*0.051; *P* = 0.010) and marriage (*β =* 0.066; *P* = 0.007) had indirect effects on practice.

**Conclusions:**

Cardiovascular health care personnel exhibited suboptimal knowledge, positive attitudes, and relatively inactive practices regarding coronary CTA and AI-assisted diagnosis. Targeted educational efforts are needed to enhance knowledge and support the integration of AI into clinical workflows.

Cardiovascular diseases remain a major cause of morbidity and mortality worldwide, accounting for approximately 17.9 million deaths annually, representing about 31% of all global deaths [1]. They include various conditions, including coronary artery disease, peripheral arterial disease, and cerebrovascular disease [2], with coronary artery disease being one of the most prevalent and severe forms [3]. Coronary artery disease is primarily caused by atherosclerosis, which involves the accumulation of plaque within the arterial walls, leading to the narrowing of coronary arteries (*i.e.* stenosis). This process can reduce blood flow, potentially resulting in myocardial infarction or sudden cardiac death. Early detection of coronary stenosis is essential for improving patient outcomes. Traditionally, invasive coronary angiography has been considered the gold standard for detecting coronary stenosis, but it carries risks like bleeding and vascular injury [4]. Non-invasive alternatives, such as stress testing and computed tomography angiography (CTA), are also widely used. CTA, in particular, offers detailed visualisation of the coronary arteries, making it a valuable non-invasive imaging technique for assessing stenosis and plaque burden [5,6]. However, challenges such as variability in interpretation and the need for operator expertise remain significant, reflecting the current clinical need for more accurate and efficient diagnostic methods.

Over the past decade, coronary CTA has advanced substantially, becoming a cornerstone in the non-invasive evaluation of coronary artery disease. It allows for the detailed visualisation of the coronary anatomy, including the lumen, wall, and plaque characteristics. Technological improvements, such as dual-source CT scanners and iterative reconstruction techniques, have enhanced the image quality and diagnostic accuracy of coronary CTA, enabling it to achieve high sensitivity and specificity. Coronary CTA has proven effective in identifying stenosis, with studies reporting sensitivity and specificity rates of 85–95% and 90–98%, respectively, depending on patient characteristics and the imaging system used [7]. Despite its effectiveness, coronary CTA's accuracy can be influenced by factors such as motion artefacts, heavy calcification, and differences in readers' expertise. These challenges result in variability in the interpretation of images, making the diagnosis of coronary artery disease inconsistent across different settings [8].

Artificial intelligence (AI) has made significant strides in various medical fields, particularly in medical imaging. In the context of coronary CTA, it has been applied to the detection, quantification, and characterisation of coronary stenosis and atherosclerotic plaque. AI algorithms, primarily based on machine learning techniques, are trained on large data sets containing annotated coronary CTA images [9], allowing them to identify patterns associated with coronary artery disease [10]. The technology has also demonstrated potential in identifying high-risk features, such as vulnerable plaques, and in reducing interpretation time, thus supporting clinical decision-making [11]. In addition to improving diagnostic accuracy, AI-based tools can support personalised treatment planning and predictive analytics, potentially optimising the management of cardiovascular diseases. However, the adoption of AI in coronary CTA faces challenges such as data privacy concerns, algorithmic bias, and transparency issues, which must be addressed to ensure safe and effective implementation [12]. As AI technologies continue to develop, their role in coronary CTA is expected to expand further, making it essential for health care professionals to stay informed about AI advancements and their implications for clinical practice.

The knowledge, attitude, and practice (KAP) model is a widely used framework for understanding health care professionals’ behaviours in adopting new technologies, including AI in coronary CTA. It posits that knowledge influences attitudes, which in turn affect practices, making it an essential approach for studying technology adoption in clinical settings. This framework has been extensively applied in health care to assess professionals’ understanding, perceptions, and use of medical technologies across various specialities, including cardiovascular imaging. By applying the KAP model, researchers can identify specific barriers to AI adoption, such as lack of knowledge about AI capabilities, concerns over algorithm accuracy, or negative attitudes influenced by ethical issues like data privacy and bias. Additionally, KAP surveys help highlight potential facilitators for enhancing adoption rates, such as training programmes, professional development opportunities, or increased awareness of AI's benefits. In the field of coronary CTA, KAP studies have focussed on clinicians' knowledge of imaging modalities, their attitudes towards non-invasive diagnostic tools, and their practices in applying these tools to patient care. However, limited attention has been paid to how these factors specifically influence the adoption of AI technologies in coronary CTA. Understanding health care providers’ knowledge levels and attitudes toward AI can provide crucial insights for developing targeted interventions to improve AI integration in clinical workflows. This approach is particularly relevant given the rapid advancement of AI technologies and the increasing demand for efficient diagnostic solutions in cardiovascular care [13–15]. By assessing health care professionals' KAP regarding AI-assisted coronary CTA, we aimed to uncover gaps in knowledge or attitudes that may hinder AI adoption and inform strategies for effective implementation. These findings can add to the evidence based for future, ultimately supporting the widespread integration of AI in cardiovascular diagnostics and enhancing patient outcomes.

## METHODS

### Study design and participants

We conducted this cross-sectional study from 1 July 2024 to 1 August 2024 at Tsinghua University Hospital in Beijing, China, a major urban centre known for its advanced health care infrastructure and educational institutions. The Tsinghua University Science and Technology Ethics Committee (Medicine) gave ethical approval for this study (Project No.: THU01-20240092). For the purposes of this study, we focussed on and included health care professionals (physicians and nurses) working in cardiovascular-related departments.

### Questionnaire distribution

We distributed the electronic questionnaires through WeChat workgroups using a survey created on the Questionnaire Star platform, which generated a QR code. Questionnaire Star is a widely used online survey tool in China, known for its secure data handling and user-friendly interface, enabling efficient data collection in health care research [16,17]. Participants accessed and completed the questionnaire by scanning the QR code with WeChat. We obtained the informed consent from all participants before questionnaire completion. After scanning the QR code, participants were prompted to fill out an informed consent form before proceeding with the questionnaire. If a participant chose not to provide consent, the questionnaire would automatically close, ensuring that only those who agreed continued with the survey. To ensure the quality and completeness of responses, each IP address was limited to one submission, and participation was voluntary. We reviewed the questionnaires for completeness, internal consistency, and response validity.

### Questionnaire overview

We designed the questionnaire based on relevant literature [18–20]. Additionally, we conducted a pilot test involving 30 participants, and the overall Cronbach's α coefficient for the questionnaire was 0.904, indicating high reliability.

The final questionnaire, written in Chinese, consisted of 40 items across four dimensions: 11 items on basic demographic information, 11 items in the knowledge dimension (with item 9 serving as a trap question to exclude invalid responses), 8 items in the attitude dimension, and 10 items in the practice dimension. For statistical analysis, we assigned scores based on the number of response options for each item. For example, in the knowledge dimension, we scored responses as 2 for ‘Very familiar’, 1 for ‘Somewhat familiar’, and 0 for ‘Unfamiliar’, with a total score range of 0–20. In the attitude dimension, scores ranged from 5 (‘Strongly agree’) to 1 (‘Strongly disagree’), with a total score range of 8–40. For the practice dimension, scores ranged from 5 (‘Always’) to 1 (‘Never’), with a total score range of 10–50. A score above 70% of the maximum in each section was considered indicative of adequate knowledge, a positive attitude, and proactive practices [21].

### Sample size calculation

We calculated our sample size using the standard formula for the minimum sample size required in cross-sectional studies:

n = (*Z*^2^ × *P* × (1 − *P*)) / *E*^2^

Where *n* is the required sample size, *Z* the *z*-value corresponding to the desired confidence level (for a 95% confidence level, *Z* = 1.96), *P* the estimated proportion of the population (commonly assumed to be 0.5 when unknown), and *E* the margin of error (typically set at 0.05).

For a 95% confidence level, with *P* = 0.5 and E = 0.05, the formula calculates as:

n = (1.96^2^ × 0.5 × (1 − 0.5)) / 0.05^2^ = 384.16

We set the effective questionnaire recovery rate at 80% and estimated that we would require a minimum of 480 questionnaires for analysis.

### Statistical methods

We conducted the data analysis using *R*, version 4.3.2 (R Core Team, Vienna, Austria). We applied normality tests to each dimension's scores. We used means (x̄) and standard deviations (SDs) for normally distributed data and medians (MD) with interquartile ranges (IQRs) for non-normal data. We assessed group differences using the *t*-test or Wilcoxon-Mann-Whitney test for two groups, and ANOVA or Kruskal-Wallis test for three or more groups, depending on normality and variance homogeneity. We used Spearman’s correlation coefficients (*r*) for correlation analysis based on data distribution. Additionally, we performed univariate and multivariate regression analyses, including variables with *P* < 0.1 from univariate analysis in the multivariate model. We used a structural equation model (SEM) to examine whether attitudes mediate the relationship between knowledge and practice. Goodness-of-fit indices included the root mean square error of approximation (RMSEA), the standardised root mean square residual (SRMR), the Tucker-Lewis index (TLI), and the comparative fit index (CFI), with thresholds of RMSEA<0.08, SRMR<0.08, TLI>0.8, and CFI>0.8. If these were not met, we applied path analysis. For all analyses, we considered *P* < 0.05 to be statistically significant.

## RESULTS

We initially received 520 questionnaires. After excluding 24 with a response time shorter than 80 seconds, 496 valid questionnaires remained for analysis. The internal consistency of the overall scale and its subscales in the formal experiment was satisfactory. The overall Cronbach’s α coefficient was 0.8965, and was otherwise 0.8890 for the knowledge section, 0.7408 for the attitude section, and 0.9035 for the practice section. The KMO value for the overall scale was 0.9156, indicating good sampling adequacy.

### Demographic information

Among the 496 participants, 290 (58.5%) were female, 234 (47.2%) were aged 36–45 years, 261 (52.6%) held a bachelor’s degree, 202 (40.7%) worked in the radiology department, and 162 (32.7%) were from the cardiology department (**Table 1**). Additionally, 364 (73.4%) had been employed for ≥10 years, 115 (23.2%) were slightly dissatisfied with their current job, and 367 (74.0%) worked 1–4 night shifts per month. The mean score for knowledge was 13.87 (SD = 4.96, possible range = 0–20), 28.25 (SD = 4.35, possible range = 8–40) for attitude, and 31.67 (SD = 8.23, possible range = 10–50) for practice. Knowledge scores showed significant variation by gender (*P* < 0.001), age (*P* = 0.045), marital status (*P* = 0.006), education level (*P* < 0.001), workplace (*P* < 0.001), department (*P* < 0.001), professional title (*P* = 0.015), job satisfaction (*P* < 0.001), employment status (*P* < 0.001), and frequency of night shifts per month (*P* < 0.001). Attitude scores significantly varied by marital status (*P* = 0.025), workplace (*P* = 0.002), department (*P* < 0.001), and job satisfaction (*P* < 0.001). Practice scores differed significantly by gender (*P* < 0.001), workplace (*P* < 0.001), department (*P* < 0.001), job satisfaction (*P* < 0.001), employment status (*P* < 0.001), and frequency of night shifts per month (*P* < 0.001).

**Table 1:** Demographic information

		Knowledge		Attitude		Practice	
	**n (%) of participants**	**MD (IQR)**	***P-*value**	**MD (IQR)**	***P-*value**	**MD (IQR)**	***P*-value**
**Total score**	496 (100.0)	13.87 (4.96)		28.25 (4.35)		31.67 (8.23)	
**Gender**			<0.001		0.366		<0.001
Male	206 (41.5)	15.03 (4.44)		28.06 (4.54)		33.33 (7.86)	
Female	290 (58.5)	13.05 (5.16)		28.38 (4.22)		30.50 (8.29)	
**Age in years**			0.045		0.212		0.233
≤35	90 (18.1)	12.76 (4.96)		28.64 (4.81)		32.72 (8.05)	
36–45 y	234 (47.2)	14.30 (4.62)		27.93 (4.24)		31.86 (8.47)	
>45	172 (34.7)	13.87 (5.35)		28.47 (4.25)		30.87 (7.95)	
**Marital status**			0.006		0.025		0.999
Married	438 (88.3)	14.08 (4.97)		28.08 (4.31)		31.64 (8.30)	
Other	58 (11.7)	12.34 (4.67)		29.48 (4.57)		31.93 (7.74)	
**Educational level**			<0.001		0.417		0.294
College diploma or below	25 (5.0)	10.12 (5.40)		28.48 (4.90)		29.00 (8.13)	
Bachelor's degree	261 (52.6)	14.62 (4.79)		27.92 (3.77)		31.77 (7.94)	
Master's degree or above	210 (42.3)	13.39 (4.88)		28.62 (4.92)		31.87 (8.56)	
**Work institution**			<0.001		0.002		<0.001
Public tertiary hospital	200 (40.3)	15.15 (4.52)		28.98 (4.31)		34.05 (7.19)	
Public secondary hospital	260 (52.4)	13.08 (5.02)		27.70 (4.27)		29.88 (8.45)	
Public community health centre or private hospital	36 (7.3)	12.53 (5.46)		28.11 (4.71)		31.36 (8.83)	
**Department**			<0.001		<0.001		<0.001
Cardiology	162 (32.7)	15.06 (4.35)		28.17 (4.09)		33.06 (6.55)	
Cardiothoracic surgery	13 (2.6)	11.23 (4.36)		28.15 (3.76)		32.62 (5.88)	
Neurology	13 (2.6)	14.46 (2.73)		24.69 (3.09)		32.31 (8.53)	
Neurosurgery	8 (1.6)	11.88 (4.36)		25.00 (4.87)		29.75 (5.50)	
Radiology	202 (40.7)	15.38 (4.11)		29.22 (4.78)		34.28 (7.80)	
Surgery	19 (3.8)	12.63 (4.62)		25.26 (3.38)		30.00 (7.64)	
Other departments	79 (15.9)	8.43 (4.73)		27.56 (3.21)		22.49 (6.56)	
**Years of working experience**			0.669		0.343		0.082
≤5	58 (11.7)	13.81 (3.65)		27.71 (4.96)		33.05 (7.45)	
5–10	74 (14.9)	13.81 (5.03)		28.23 (4.34)		33.05 (8.37)	
>10	364 (73.4)	13.90 (5.14)		28.34 (4.26)		31.17 (8.28)	
**Professional title**			0.015		0.201		0.225
Junior	67 (13.5)	12.28 (5.07)		27.79 (4.58)		30.24 (8.25)	
Intermediate	255 (51.4)	14.00 (5.02)		28.02 (4.20)		31.96 (8.19)	
Senior	174 (35.1)	14.29 (4.75)		28.75 (4.46)		31.79 (8.25)	
**Job satisfaction**			<0.001		<0.001		<0.001
Very satisfied	97 (19.6)	14.26 (4.46)		29.60 (4.23)		34.22 (8.86)	
Fairly satisfied	180 (36.3)	12.92 (5.29)		29.17 (4.01)		32.06 (8.67)	
Neutral	104 (21.0)	12.83 (5.10)		27.72 (4.13)		30.01 (7.10)	
Slightly dissatisfied	115 (23.2)	15.99 (3.96)		26.14 (4.36)		30.43 (7.37)	
**Employment status**			<0.001		0.409		<0.001
Permanent	425 (85.7)	14.35 (4.76)		28.31 (4.42)		32.22 (8.01)	
Contractual	57 (11.5)	10.47 (5.38)		27.77 (4.17)		27.54 (8.35)	
Human resources agency	14 (2.8)	13.21 (4.12)		28.29 (3.10)		31.71 (9.83)	
**Night shifts per month**			<0.001		0.126		<0.001
None	78 (15.7)	11.33 (5.11)		29.13 (3.94)		26.33 (8.71)	
1–4	367 (74.0)	14.28 (4.95)		28.16 (4.41)		32.83 (7.76)	
≥5	51 (10.3)	14.84 (3.53)		27.51 (4.41)		31.49 (7.60)	

IQR – interquartile range, MD – median, SD – standard deviation, x̄ – mean

### KAP analysis

In the knowledge section (Table S1 in the **Online Supplementary Document**), the three items with the highest proportion of ‘Unfamiliar’ responses were as follows:

- ‘Compared to traditional denoising techniques such as iterative algorithms, which require projection domain data, deep learning methods can directly denoise and optimise reconstructed CT images without relying on projection data, resolving the challenge of users being unable to access intermediate projection data from CT scanners’ (K11, 25.4%).
- ‘AI-assisted diagnosis reduces patient radiation exposure while maintaining image quality, ensuring that clinical requirements are met’ (K10, 21.6%).
- ‘The deep learning neural network algorithm can directly calculate 3D models, automating image preprocessing, vessel segmentation, lesion detection, and classification, which reduces radiologists' workload and enhances efficiency’ (K7, 19.4%).

Regarding attitudes (Table S2 in the **Online Supplementary Document**), 13.7% agreed that AI-assisted diagnosis might affect patients' trust in diagnostic results, leading to distrust or complaints about doctors (A5), while 17.5% agreed that AI diagnosis, despite its auxiliary role, still carries ethical risks and conflicts with humanistic principles (A6). Additionally, 16.5% of participants strongly agreed and 31.2% agreed that they inherently do not trust AI-assisted diagnosis results, but are required to use it (A8).

In terms of practice (Table S3 in the **Online Supplementary Document**), 26.2% of participants reported never discussing system improvements with AI engineers (P7), 23.2% never explained the diagnostic process and AI system use to patients (P4), and 21.8% never manually re-diagnosed after using AI to ensure accuracy (P6).

### Correlations between KAP

Correlation analysis showed significant positive correlations between knowledge and practice (*r* = 0.358; *P* < 0.001), and between attitude and practice (*r =* 0.489; *P* < 0.001), but no significant correlation between knowledge and attitude (*r =* 0.043; *P* = 0.334) (Table S4 in the **Online Supplementary Document**).

### Univariate and multivariate analysis of KAP dimensions

We grouped the participants based on their median scores for KAP dimensions. The number of participants scoring above the cut-off was 269 (54.23%) for knowledge, 285 (57.46%) for attitude, and 265 (53.43%) for practice (Table S5 in the **Online Supplementary Document**). Multivariate logistic regression revealed that having a bachelor’s degree (odds ratio (OR) = 7.737; 95% confidence interval (CI) = 1.916–31.244, *P* = 0.004), a master’s degree or above (OR = 4.804; 95% CI = 1.170–19.724, *P* = 0.029), working in the cardiology department (OR = 8.388; 95% CI = 3.549–19.821, *P* < 0.001) or the radiology department (OR = 9.747; 95% CI = 4.314–22.022, *P* < 0.001), and having neutral (OR = 0.394; 95% CI = 0.209–0.743, *P* = 0.004) or slight job dissatisfaction (OR = 0.535; 95% CI = 0.297–0.965, *P* = 0.038) were independently associated with knowledge (Table S6 in the **Online Supplementary Document**). Meanwhile, being married (OR = 0.235; 95% CI = 0.106–0.519, *P* < 0.001), working in the cardiology department (OR = 2.903; 95% CI = 1.454–5.794, *P* = 0.003) or the radiology department (OR = 3.180; 95% CI = 1.647–6.142, *P* = 0.001), having 5–10 years of working experience (OR = 4.647; 95% CI = 1.882–11.472, *P* = 0.001), having more than 10 years of working experience (OR = 2.863; 95% CI = 1.271–6.448, *P* = 0.011), or being very satisfied (OR = 6.651; 95% CI = 3.448–12.829, *P* < 0.001), fairly satisfied (OR = 5.432; 95% CI = 3.065–9.628, *P* < 0.001), or neutral about current job (OR = 2.147; 95% CI = 1.189–3.877, *P* = 0.011) were independently associated with attitude (Table S7 in the **Online Supplementary Document**). Knowledge score (OR = 1.107; 95% CI = 1.046–1.171, *P* < 0.001), attitude score (OR = 1.237; 95% CI = 1.162–1.317, *P* < 0.001), being from any department (OR>1; *P* < 0.05), and being very satisfied (OR = 5.317; 95% CI = 2.405–11.756, *P* < 0.001) or fairly satisfied with current job (OR = 5.144; 95% CI = 2.627–10.072, *P* < 0.001) were independently associated with practice (Table S8 in the **Online Supplementary Document**).

### SEM results

**Figure 1:**
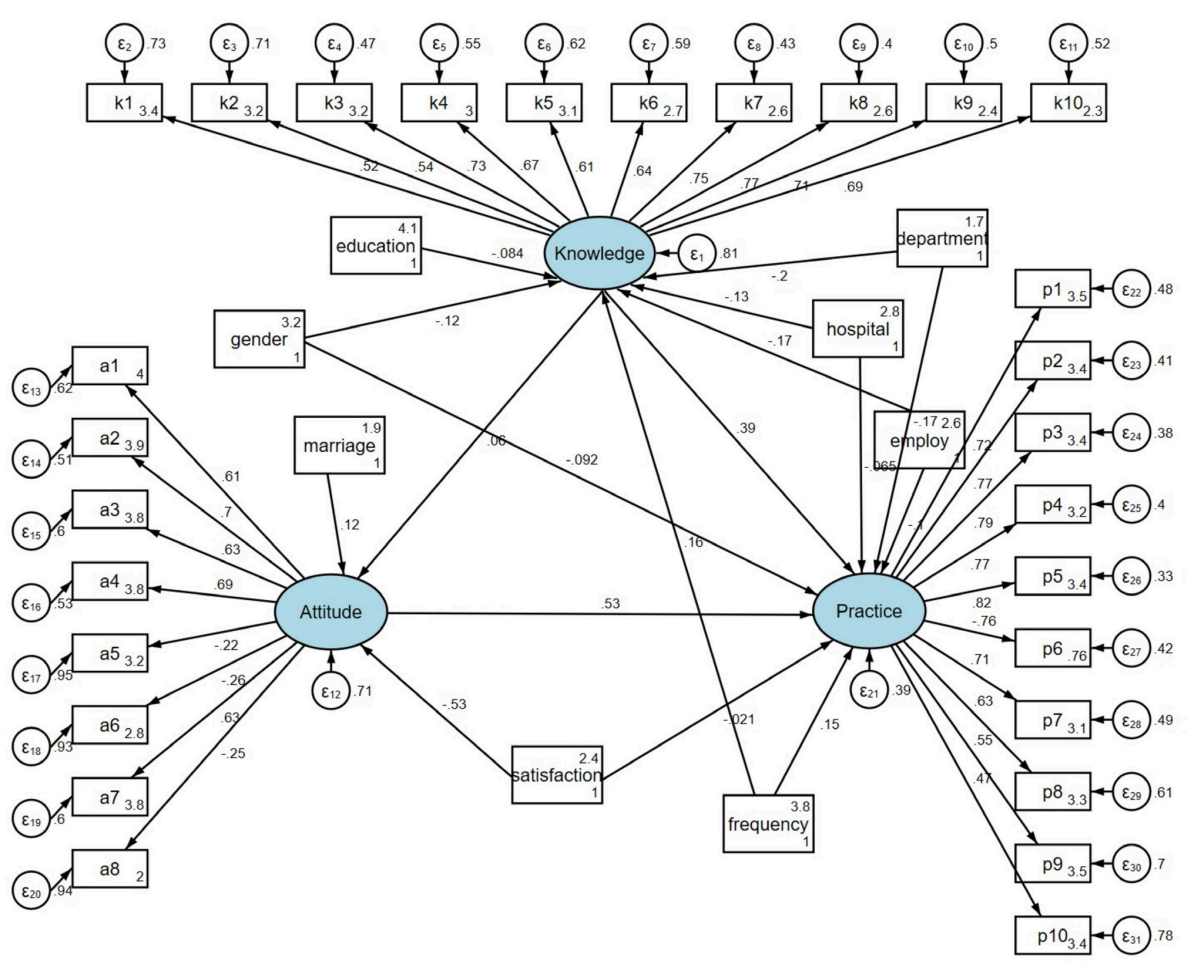
SEM analysis results.

The fit indices for the SEM (RMSEA = 0.079, SRMR = 0.095, TLI = 0.739, CFI = 0.759) exceeded the respective threshold values, indicating that the data adequately fit the model (Tables S9 and S10 in the **Online Supplementary Document**). Analysis of direct and indirect effects revealed that gender (β = −0.121; *P* = 0.009), workplace (*β = −*0.133; *P* = 0.004), department (*β = −*0.197; *P* < 0.001), employment status (*β = −*0.166; *P* < 0.001), and night shift frequency (*β =* 0.163; *P* < 0.001) directly affected knowledge; marriage (*β =* 0.124; *P* = 0.006) and job satisfaction (*β = −*0.528; *P* < 0.001) directly influenced attitude. Knowledge (*β =* 0.389; *P* < 0.001), attitude (*β =* 0.533; *P* < 0.001); and gender (*β = −*0.092; *P* = 0.010) directly affected practice. Additionally, gender (*β = −*0.051; *P* = 0.010) and marriage (*β =* 0.066; *P* = 0.007) indirectly affected practice (**Figure 1**; Table S11 in the **Online Supplementary Document**).

## DISCUSSION

Cardiovascular health care personnel exhibited limited knowledge, generally positive attitudes, and inactive practices regarding coronary CTA and AI-assisted diagnosis. Targeted educational programmes and hands-on training are recommended to enhance knowledge and encourage the active use of AI-assisted diagnostic tools in clinical settings, potentially improving both diagnostic accuracy and health care efficiency.

We found that cardiovascular health care personnel exhibited suboptimal knowledge, generally positive attitudes, and inactive practices regarding coronary CTA and AI-assisted diagnosis. These findings align with other research targeting medical professionals, including physicians, radiologists, and medical students. This consistently demonstrates that limited knowledge of AI is associated with greater concerns about its accuracy and the potential threat of being replaced by technology. Conversely, those with more extensive AI training and understanding express fewer concerns about job displacement and instead show greater interest in learning how to integrate AI into their practice [22,23]. The observed suboptimal knowledge suggests that inadequate exposure to AI may be contributing to similar fears and uncertainties among health care workers, which could hinder the broader adoption of AI-assisted tools in clinical settings.

In line with other studies, we found AI knowledge to be a key factor influencing health care professionals’ confidence in AI systems [24,25]. Our findings demonstrated that males, radiology department staff, and those with bachelor’s or higher degrees had significantly higher knowledge levels. This comfort with AI likely stems from increased exposure and familiarity with the technology during their clinical training. However, the significant gender difference in knowledge highlights a potential disparity in access to AI education, which should be addressed to ensure equitable learning opportunities across all health care sectors.

While knowledge significantly correlated with practice, it did not correlate with attitudes in our study. This discrepancy could be explained by the fact that, despite a lack of understanding, health care workers may still recognise the potential benefits of AI in improving diagnostic accuracy and workflow efficiency. Studies involving radiologists have shown that, although AI’s diagnostic potential is widely acknowledged, professionals remain cautious about the accuracy of AI-assisted systems [26,27]. This cautious optimism may be reflected in the generally positive attitudes observed in our study, even among participants with lower knowledge levels.

The practice dimension results in our study align with research demonstrating a gap between interest in AI technologies and their actual use in clinical practice [28,29]. While most respondents expressed interest in learning about AI, only a small portion actively engaged with AI systems in their daily workflows. This ‘interest-practice gap’ has been similarly observed in studies involving radiologists and medical students, where there is enthusiasm for AI, but limited integration into practice due to insufficient training or system reliability concerns [30,31]. A significant number of respondents indicated that they seldom re-diagnose manually after using AI, reflecting a reliance on AI-based systems over traditional methods. This highlights a critical need for practical, hands-on training that focusses not only on how AI functions but also on integrating AI tools seamlessly into clinical routines.

Multivariate analysis and SEM results revealed that gender, job satisfaction, work institution, and department were strong predictors of KAP outcomes. Specifically, higher knowledge and practice scores among males, those working in radiology departments, and individuals in public tertiary hospitals suggest that these groups have greater access to AI technologies and more opportunities to apply them in clinical settings. Radiologists, in particular, often have early exposure to AI, which has been linked to reduced concerns about being replaced by machines and greater confidence in AI’s potential to improve diagnostic accuracy [32]. On the other hand, variables such as job satisfaction played a notable role across all KAP dimensions, with those expressing higher job satisfaction also demonstrating more positive attitudes and better practices. Conversely, those with lower job satisfaction may feel less inclined to invest time in learning new technologies, leading to lower AI usage in practice. Interventions to improve job satisfaction, such as offering continuing education on AI and creating a supportive work environment, could therefore enhance AI adoption.

The negative relationships identified in the SEM analysis indicate potential structural barriers affecting KAP outcomes. Lower knowledge and practice scores among certain groups, such as females, non-tertiary hospital staff, and those outside radiology departments, suggest that disparities in training opportunities and access to AI resources may contribute to these differences. Furthermore, reduced job satisfaction appears to be associated with less favourable attitudes and practices, implying that the workplace environment and professional development play crucial roles in the adoption of AI. Addressing these barriers through targeted interventions, such as increasing access to AI training and fostering supportive work environments, could enhance both the knowledge and practical application of AI technologies among health care professionals.

The results from the knowledge distribution further underscore the need for improved education on AI functionality. Many participants were unclear about critical aspects of AI systems, particularly in understanding how AI integrates into coronary CTA workflows. This mirrors other studies, where medical professionals expressed a lack of familiarity with AI's specific applications and limitations [33,34]. For example, a large portion of respondents did not fully grasp the AI-assisted system's capabilities in noise reduction and image optimisation, which may hinder their confidence in using AI tools. To address these knowledge gaps, targeted AI education programmes, particularly focussing on real-world applications and case studies, could be introduced, especially for departments like radiology and cardiology, where AI is likely to be implemented more frequently.

We found that most health care personnel had a generally favourable view of AI, with many seeing its potential to improve efficiency. However, a significant proportion still harboured concerns about AI's accuracy and ethical implications, a finding consistent with research on medical students and radiologists who, despite recognising AI's benefits, remain cautious about its reliability and potential impact on the doctor-patient relationship [35,36]. Providing transparency in AI decision-making processes and demonstrating AI's accuracy through clinical evidence could alleviate these concerns.

Regarding inactive practices, the reluctance to engage fully with AI systems points to an opportunity for more structured integration of AI into clinical workflows. Institutions should implement mandatory AI training workshops, offer real-time AI system demonstrations, and encourage more collaboration between clinicians and AI developers. These hands-on experiences would help bridge the gap between interest in AI and actual practice, as professionals would feel more confident in their ability to use these systems effectively. Specific strategies include expanding access to AI-focussed continuing education programmes tailored to different professional levels. For radiologists and cardiologists, who are more likely to work with AI technologies, advanced training on AI integration into diagnostic processes could improve both knowledge and practice [37,38]. Additionally, fostering collaborative workshops between AI engineers and health care professionals would provide opportunities for real-time troubleshooting and customisation of AI systems to better fit clinical needs. These workshops could also address the trust gap by offering detailed insights into how AI algorithms function and demonstrating their clinical efficacy. Lastly, institutions should consider offering incentives, such as career development opportunities or certification programmes, to motivate health care personnel to actively engage with AI tools [39,40].

This study has several limitations. First, cross-sectional design limits the ability to establish causality between demographic factors and KAP outcomes. Second, the survey was conducted at a single institution, which may limit the generalisability of the findings to other settings or regions. Third, self-reported data may introduce bias, as participants might overestimate or underestimate their knowledge, attitudes, or practices related to coronary CTA and AI-assisted diagnosis.

## CONCLUSIONS

Cardiovascular health care personnel demonstrated suboptimal knowledge, positive attitudes, and inactive practices regarding CTA and AI-assisted diagnosis. These findings suggest the need for targeted educational interventions to enhance health care workers' knowledge and improve their practical application of AI-assisted diagnostic tools, which may ultimately lead to better integration of AI in clinical practice.

## Additional Material


Online Supplementary Document
