# Supplementary material for: Knowledge, attitudes, and practices of cardiovascular health care personnel regarding coronary CTA and AI-assisted diagnosis: a cross-sectional study
Source: J Glob Health. 2025 Jul 4;15:04103. doi: 10.7189/jogh.15.04103 (PMC12231367; doi:10.7189/jogh.15.04103)
Supplement: Online Supplementary Document [file jogh-15-04103-s001.pdf]

Supplement to: Jiang S, Ma L, Pan K, Zhang H. Knowledge, attitudes, and practices of cardiovascular healthcare personnel regarding coronary CTA and AI-assisted diagnosis: a cross-sectional study. J Glob Health. 2025;15:04103.

Table S1. Levels of knowledge among participants regarding coronary CTA and AI-assisted diagnostic systems

	Familiar	Somewhat familiar	Unfamiliar
**1. Coronary CTA requires the injection of iodinated contrast agents.**	314(63.3%)	161(32.5%)	21(4.2%)
**2. Coronary CTA can determine the degree of coronary artery stenosis, lesion location, and whether coronary calcification and plaques are present.**	310(62.5%)	152(30.6%)	34(6.9%)
**3. Image evaluation in coronary CTA typically requires the use of CT reconstruction software.**	304(61.3%)	147(29.6%)	45(9.1%)
**4. The general sensitivity of coronary CTA in diagnosing coronary artery disease is approximately 70%, with a specificity of up to 90%.**	279(56.2%)	163(32.9%)	54(10.9%)
**5. Contraindications for coronary CTA include severe renal insufficiency, pregnancy, and iodine allergy.**	293(59.1%)	163(32.9%)	40(8.1%)
**6. The artificial intelligence-assisted diagnostic system for coronary CTA operates by processing data through a computer, instantly displaying curved images of each coronary vessel, labelling coronary segments by name, diagnosing and measuring plaques, and aiding clinicians in detecting coronary lumen stenosis. This significantly improves the diagnostic efficiency and accuracy of coronary CTA.**	230(46.4%)	198(39.9%)	68(13.7%)
**7. The deep learning neural network algorithm can directly calculate 3D models, completing the full workflow of image preprocessing, vessel segmentation and extraction, vessel integrity repair, automatic vessel labelling, intelligent lesion detection, and lesion classification. Its display through the software interface plays a positive role in reducing the workload of radiologists and enhancing work efficiency.**	228(46%)	172(34.7%)	96(19.4%)
**8. One application of artificial intelligence in coronary CTA lies in its ability to remove complex noise from low-dose CT images through feature learning and mapping of deep learning neural network algorithms.**	236(47.6%)	165(33.3%)	95(19.2%)
**10. In addition to separating noise from images via the learning of noise models from low-dose CT images, artificial intelligence assistance reduces patient radiation exposure while ensuring that image quality still meets clinical requirements.**	209(42.1%)	180(36.3%)	107(21.6%)
**11. Compared to traditional denoising techniques like iterative algorithms, which require**	204(41.1%)	166(33.5%)	126(25.4%)

projection domain data, deep learning methods can directly denoise and optimise reconstructed CT images without relying on projection data. This also resolves the challenge of users being unable to access intermediate projection data from CT scanners.

Table S2. Attitudes of participants toward coronary CTA and AI-assisted diagnosis

	Strongly Agree	Agree	Neutral	Disagree	Strongly Disagree
**1. You are highly interested in learning about coronary CTA and AI-assisted diagnosis.**	165(33.3%)	156(31.5%)	108(21.8%)	41(8.3%)	26(5.2%)
**2. You believe that, compared to the drawbacks of AI assistance, the advantages of streamlining the traditional workflow of coronary CTA and improving efficiency are more deserving of promotion.**	158(31.9%)	173(34.9%)	88(17.7%)	42(8.5%)	35(7.1%)
**3. You personally have a high level of trust in the diagnostic results provided by the AI-assisted diagnostic system for coronary CTA.**	129(26%)	183(36.9%)	97(19.6%)	51(10.3%)	36(7.3%)
**4. You believe that patients also place significant trust in the diagnostic results from the AI-assisted coronary CTA system.**	130(26.2%)	172(34.7%)	102(20.6%)	57(11.5%)	35(7.1%)
**5. You think that if patients were aware that, although a doctor is involved throughout the process, AI assistance is used for diagnostic, it would to some extent affect their trust in the results, lead to doubts about the physician's personal abilities, and potentially result in complaints from patients.**	28(5.6%)	68(13.7%)	60(12.1%)	299(60.3%)	41(8.3%)
**6. You consider that, despite physician involvement, the current role of AI diagnosis in clinical settings is merely auxiliary, yet still presents considerable ethical risks. Relying on machines for diagnostic decisions is not aligned with humanistic principles.**	35(7.1%)	87(17.5%)	46(9.3%)	280(56.5%)	48(9.7%)
**7. You believe that since the AI system is only supplementary, it is essential to strictly adhere to the operational guidelines and avoid all potential pitfalls to ensure the reliability and credibility of the results.**	155(31.2%)	184(37.1%)	71(14.3%)	47(9.5%)	39(7.9%)
**8. At your core, you do not trust AI-assisted diagnostic results. You only use it because your colleagues are utilising it and your superiors require it. You believe that only results that have been manually reviewed are trustworthy.**	82(16.5%)	155(31.2%)	106(21.4%)	119(24%)	34(6.9%)

Table S3. Practices of participants regarding coronary CTA and AI-assisted diagnosis

	Strongly Agree	Agree	Neutral	Disagree	Strongly Disagree
**1. You frequently study knowledge related to coronary CTA and AI-assisted diagnosis.**	109 (22%)	131(26.4%)	84(16.9%)	103(20.8%)	69(13.9%)
**2. You often discuss issues concerning the interpretation of coronary CTA diagnostic results with radiologists.**	107(21.6%)	133(26.8%)	80(16.1%)	92(18.5%)	84(16.9%)
**3. You regularly use the AI-assisted system to aid in the diagnosis of coronary CTA results.**	105(21.2%)	146(29.4%)	79(15.9%)	62(12.5%)	104(21%)
**4. You frequently explain the diagnostic process of coronary CTA and the use of the AI-assisted system in detail to patients.**	96(19.4%)	109(22%)	86(17.3%)	90(18.1%)	115(23.2%)
**5. You often share your experiences and precautions regarding the use of the AI-assisted system with colleagues to prevent potential misoperations by yourself or other doctors.**	100(20.2%)	130(26.2%)	77(15.5%)	86(17.3%)	103(20.8%)
**6. You regularly re-evaluate the diagnostic results manually after using the AI-assisted system for coronary CTA before ensuring accuracy and uploading the results to the system for patients.**	105(21.2%)	134(27%)	76(15.3%)	73(14.7%)	108(21.8%)
**7. You frequently discuss feasible system improvement solutions with AI-assisted system engineers.**	83(16.7%)	124(25%)	68(13.7%)	91(18.3%)	130(26.2%)
**8. For special critical patients, since the AI-assisted system can significantly save time, you are relatively more inclined to use AI-assisted diagnosis from the perspective of patient benefit to save time.**	113(22.8%)	149(30%)	83(16.7%)	71(14.3%)	80(16.1%)
**9. Although AI-assisted diagnosis can shorten diagnostic time, there is still the risk of adverse reactions in patients due to the contrast agent, unrelated to AI-assisted diagnosis, and you will still thoroughly inform patients of the associated risks.**	133(26.8%)	162(32.7%)	83(16.7%)	56(11.3%)	62(12.5%)
**10. AI-assisted diagnosis can greatly enhance work efficiency, and from a health economics perspective, it is necessary to strongly promote the application of the current technology in the field of coronary CTA diagnosis.**	134(27%)	166(33.5%)	76(15.3%)	59(11.9%)	61(12.3%)

Table S4. Spearman correlation coefficients between knowledge, attitudes, and practice

	Knowledge		Attitudes		Practice	
	r	***P*-value**	*r*	***P*-value**	*r*	***P*-value**
**Knowledge**	1.000					
**Attitudes**	0.043	0.334	1.000			
**Practice**	0.358	<0.001	0.489	<0.001	1.000	

Table S5. Cut-off Values for Univariate and Multivariate Logistic Regression Analysis

**Cut-off value: median**	N (%)
Total score for the Knowledge Dimension	
**Ksum>=15**	269(54.23%)
**Ksum<=14**	227(45.77%)
Total score for the Attitude Dimension	
**Asum>=28**	285(57.46%)
**Asum<=27**	211(42.54%)
Total score for the Practice Dimension	
**Psum>=32**	265(53.43%)
**Psum<=31**	231(46.57%)

Table S6. Multivariate logistic regression analysis – knowledge dimension

	**Univariate analysis**		**Multivariate analysis**	
	**OR (95%CI)**	***P*-value**	**OR(95%CI)**	***P*-value**
**Gender**				
Male				
Female	0.520 (0.360,0.748)	0.001	0.739 (0.478,1.144)	0.175
**Age (in years)**				
≤35				
36–45	1.847 (1.133,3.032)	0.014	1.320 (0.661,2.633)	0.431
>45	1.539 (0.923,2.583)	0.100	1.048 (0.486,2.260)	0.904
**Marital status**				

Married	1.950 (1.122,3.448)	0.019	1.192 (0.557,2.549)	0.651
Other				
**Educational level**				
College diploma or below				
Bachelor's degree	8.184 (3.012,28.666)	<0.001	7.737 (1.916,31.244)	0.004
Master's degree or above	5.351 (1.957,18.816)	0.003	4.804 (1.170,19.724)	0.029
**Work Institution**				
Public tertiary hospital	0.459 (0.313,0.669)	<0.001	0.827 (0.323,2.118)	0.693
Public secondary hospital	0.471 (0.228,0.965)	0.040	0.564 (0.225,1.412)	0.221
Public community health centre or private hospital				
**Department**				
Cardiology	12.026 (6.092,25.743)	<0.001	8.388 (3.549,19.821)	<0.001
Cardiothoracic surgery	1.855 (0.373,7.270)	0.400	1.257 (0.246,6.425)	0.783
Neurology	7.212 (2.049,26.603)	0.002	2.524 (0.580,10.980)	0.217
Neurosurgery	2.061 (0.278,10.357)	0.411	1.853 (0.296,11.585)	0.510
Radiology	11.162 (5.752,23.546)	<0.001	9.747 (4.314,22.022)	<0.001
Surgery	5.564 (1.846,17.136)	0.002	2.776 (0.786,9.804)	0.113
Other departments				
**Years of working experience**				
≤5				
5–10	1.114 (0.560,2.223)	0.758		
>10	1.233 (0.707,2.152)	0.459		
**Professional title**				
Junior				
Intermediate	1.498 (0.873,2.591)	0.144	0.647 (0.266,1.571)	0.336
Senior	1.947 (1.104,3.465)	0.022	0.883 (0.326,2.391)	0.806
**Job satisfaction**				
Very satisfied	0.564 (0.339,0.928)	0.025	1.051 (0.525,2.104)	0.888
Fairly satisfied	0.491 (0.278,0.859)	0.013	0.535 (0.297,0.965)	0.038
Neutral	1.534 (0.867,2.727)	0.142	0.394 (0.209,0.743)	0.004
Slightly dissatisfied				
**Employment status**				
Permanent	1.401 (0.472,4.161)	0.535	1.283 (0.327,5.039)	0.721
Contractual	0.326 (0.095,1.101)	0.069	0.765 (0.182,3.221)	0.715
Human resources agency				
**Night shifts per month**				
None	0.287 (0.135,0.595)	0.001	0.408 (0.155,1.076)	0.070
1–4 times	0.902 (0.489,1.632)	0.737	0.365 (0.163,0.817)	0.014
≥5				

Table S7. Multivariate logistic regression analysis – attitudes dimension

	Univariate analysis		Multivariate analysis	
	OR (95%CI)	*P*-value	OR (95%CI)	*P*-value
**Knowledge**	1.030 (0.994,1.068)	0.103	1.027 (0.979,1.076)	0.277
**Gender**				
Male				
Female	1.241 (0.865,1.781)	0.241		
**Age (in years)**				
⩽35				
36–45	0.733 (0.443,1.201)	0.222		
>45	0.864 (0.509,1.453)	0.583		
**Marital status:**				
Married	0.522 (0.281,0.931)	0.032	0.235 (0.106,0.519)	<0.001
Other				
**Educational level**				
College diploma or below				
Bachelor's degree	1.487 (0.650,3.428)	0.344		
Master's degree or above	1.502 (0.651,3.492)	0.338		
**Work Institution**				
Public tertiary hospital	1.776 (0.861,3.646)	0.117		
Public secondary hospital	0.937 (0.462,1.886)	0.855		
Public community health centre or private hospital				
**Department**				
Cardiology	1.530 (0.891,2.635)	0.123	2.903 (1.454,5.794)	0.003
Cardiothoracic surgery	1.259 (0.385,4.231)	0.701	1.941 (0.536,7.026)	0.312
Neurology	0.196 (0.029,0.791)	0.042	0.273 (0.051,1.471)	0.131
Neurosurgery	1.079 (0.240,4.854)	0.918	1.023 (0.203,5.158)	0.978
Radiology	2.174 (1.282,3.705)	0.004	3.180 (1.647,6.142)	0.001
Surgery	0.288 (0.077,0.874)	0.040	0.352 (0.094,1.322)	0.122
Other departments				
**Years of working experience**				
⩽5				
5–10	2.437 (1.211,4.987)	0.013	4.647 (1.882,11.472)	0.001
>10	1.841 (1.055,3.248)	0.033	2.863 (1.271,6.448)	0.011
**Professional title**				

Junior				
Intermediate	1.402 (0.817,2.411)	0.219		
Senior	1.568 (0.889,2.772)	0.120		
**Job satisfaction**				
Very satisfied	3.080 (1.761,5.475)	<0.001	6.651 (3.448,12.829)	<0.001
Fairly satisfied	2.823 (1.748,4.600)	<0.001	5.432 (3.065,9.628)	<0.001
Neutral	1.504 (0.882,2.575)	0.135	2.147 (1.189,3.877)	0.011
Slightly dissatisfied				
**Employment status**				
Permanent	0.764 (0.231,2.249)	0.634		
Contractual	0.617 (0.172,2.018)	0.435		
Human resources agency				
**Night shifts per month**				
None	1.664 (0.816,3.418)	0.162		
1–4	1.423 (0.790,2.568)	0.239		
≥5				

Table 8. Multivariate logistic regression analysis – practice dimension

	**Univariate analysis**		**Multivariate analysis**	
	**OR (95%CI)**	***P*-value**	**OR (95%CI)**	***P*-value**
**Knowledge**	1.138 (1.094,1.184)	<0.001	1.107 (1.046,1.171)	<0.001
**Attitude**	1.234 (1.172,1.299)	<0.001	1.237 (1.162,1.317)	<0.001
**Gender**				
Male				
Female	0.693 (0.482,0.993)	0.046	0.942 (0.584,1.518)	0.805
**Age (in years)**				
≤35				
36–45	0.940 (0.574,1.531)	0.803		
>45	0.747 (0.446,1.246)	0.265		
**Marital status**				
Married	1.081 (0.622,1.871)	0.782		
Other				
**Educational level**				
College diploma or below				
Bachelor's degree	1.736 (0.760,4.126)	0.196		
Master's degree or above	1.816 (0.788,4.352)	0.167		
**Work institution**				
Public tertiary hospital	1.392 (0.672,2.848)	0.367		
Public secondary hospital	0.665 (0.326,1.337)	0.254		

Public community health centre or private hospital				
**Department**				
Cardiology	12.545 (6.120,28.582)	<0.001	11.962 (4.473,31.992)	<0.001
Cardiothoracic surgery	9.074 (2.516,34.633)	0.001	11.649 (2.511,54.040)	0.002
Neurology	9.074 (2.516,34.633)	0.001	10.823 (2.251,52.037)	0.003
Neurosurgery	4.667 (0.847,22.623)	0.058	8.038 (1.408,45.898)	0.019
Radiology	14.351 (7.089,32.384)	<0.001	9.884 (3.849,25.382)	<0.001
Surgery	5.657 (1.792,18.156)	0.003	8.252 (2.071,32.880)	0.003
Other departments				
**Years of working experience**				
⩽5				
5–10	1.035 (0.513,2.084)	0.923		
>10	0.746 (0.421,1.302)	0.306		
**Professional title**				
Junior				
Intermediate	1.600 (0.933,2.764)	0.089		
Senior	1.352 (0.769,2.393)	0.296		
**Job satisfaction**				
Very satisfied	2.612 (1.502,4.607)	0.001	5.317 (2.405,11.756)	<0.001
Fairly satisfied	2.068 (1.289,3.339)	0.003	5.144 (2.627,10.072)	<0.001
Neutral	0.950 (0.554,1.626)	0.850	1.349 (0.703,2.588)	0.368
Slightly dissatisfied				
**Employment status**				
Permanent	0.714 (0.216,2.102)	0.552	0.340 (0.075,1.537)	0.161
Contractual	0.236 (0.064,0.785)	0.022	0.301 (0.062,1.466)	0.137
Human resources agency				
**Night shifts per month**				
None	0.378 (0.179,0.785)	0.010	0.400 (0.147,1.089)	0.073
1–4	1.391 (0.771,2.508)	0.271	1.728 (0.765,3.900)	0.188
⩾5				

Table S9. Fit Indices of the SEM Model

Indicators	Reference	Results
RMSEA	<0.08Good	0.079
SRMR	<0.08Good	0.095
TLI	>0.8Good	0.739
CFI	>0.8Good	0.759

Table S10. SEM Results of the Path Analysis in the Model

Indicators		Estimate	P>|z|
Knowledge			
	gender	-2.63	0.009
	education	-1.85	0.065
	hospital	-2.87	0.004
	department	-4.02	<0.001
	employ	-3.48	<0.001
	frequency	3.52	<0.001
Attitude			
	Knowledge	1.23	0.219
	gender	-1.12	0.263
	marriage	2.78	0.006
	education	-1.03	0.304
	hospital	-1.13	0.258
	department	-1.18	0.237

	satisfaction	-9.65	<0.001
	employ	-1.17	0.241
	frequency	1.17	0.241
Practice			
	Knowledge	7.16	<0.001
	Attitude	8.54	<0.001
	gender	-3.64	<0.001
	marriage	2.72	0.007
	education	-1.83	0.068
	hospital	-3.13	0.002
	department	-5.84	<0.001
	satisfaction	-7.23	<0.001
	employ	-4.28	<0.001
	frequency	5.44	<0.001

Table S11. SEM Results of the Direct and Indirect Effects Analysis in the Model

**Model paths**		Total effects		Direct Effect		Indirect effect	
		β(95%CI)	P	β(95%CI)	P	β(95%CI)	P
Knowledge							
	gender	-0.121(-0.211,-0.031)	0.009	-0.121(-0.211,-0.031)	0.009		
	educatio	-0.084(-	0.06	-0.084(-	0.06		

	n	0.173,0.005)	5	0.173,0.005)	5		
	hospital	-0.133(-0.223,-0.042)	0.004	-0.133(-0.223,-0.042)	0.004		
	department	-0.197(-0.293,-0.101)	<0.001	-0.197(-0.293,-0.101)	<0.001		
	employ	-0.166(-0.259,-0.073)	<0.001	-0.166(-0.259,-0.073)	<0.001		
	frequency	0.163(0.072,0.254)	<0.001	0.163(0.072,0.254)	<0.001		
Attitude							
	Knowledge	0.060(-0.036,0.156)	0.219	0.060(-0.036,0.156)	0.219		
	gender	-0.007(-0.020,0.006)	0.263			-0.007(-0.020,0.006)	0.263
	marriage	0.124(0.036,0.211)	0.006	0.124(0.036,0.211)	0.006		
	education	-0.005(-0.015,0.005)	0.304			-0.005(-0.015,0.005)	0.304
	hospital	-0.008(-0.022,0.006)	0.258			-0.008(-0.022,0.006)	0.258
	department	-0.012(-0.032,0.008)	0.237			-0.012(-0.032,0.008)	0.237
	satisfacti	-0.528(-	<0.0	-0.528(-	<0.0		

	on	0.635,-0.421)	01	0.635,-0.421)	01		
	employ	-0.010(-0.027,0.007)	0.241			-0.010(-0.027,0.007)	0.241
	frequency	0.010(-0.007,0.026)	0.241			0.010(-0.007,0.026)	0.241
Practice							
	Knowledge	0.421(0.306,0.536)	<0.001	0.389(0.281,0.497)	<0.001	0.032(-0.019,0.083)	0.216
	Attitude	0.533(0.411,0.655)	<0.001	0.533(0.411,0.655)	<0.001		
	gender	-0.143(-0.220,-0.066)	<0.001	-0.092(-0.161,-0.022)	0.010	-0.051(-0.090,-0.012)	0.010
	marriage	0.066(0.018,0.114)	0.007			0.066(0.018,0.114)	0.007
	education	-0.035(-0.073,0.003)	0.068			-0.035(-0.073,0.003)	0.068
	hospital	-0.121(-0.197,-0.045)	0.002	-0.065(-0.134,0.003)	0.062	-0.056(-0.095,-0.017)	0.005
	department	-0.249(-0.333,-0.166)	<0.001	-0.166(-0.242,-0.090)	<0.001	-0.083(-0.126,-0.040)	<0.001
	satisfaction	-0.302(-0.384,-0.220)	<0.001	-0.021(-0.105,0.064)	0.633	-0.282(-0.354,-0.209)	<0.001
	employ	-0.169(-	<0.0	-0.100(-	0.00	-0.070(-	0.00

		0.247,-0.092)	01	0.169,-0.030)	5	0.111,-0.029)	1
	frequenc<br>y	0.215(0.137,0.<br>292)	<0.0<br>01	0.146(0.076,0.<br>216)	<0.0<br>01	0.069(0.029,0.<br>108)	0.00<br>1
